# Supplementary material for: Functional Insights From KpfR, a New Transcriptional Regulator of Fimbrial Expression That Is Crucial for Klebsiella pneumoniae Pathogenicity
Source: Front Microbiol. 2021 Jan 21;11:601921. doi: 10.3389/fmicb.2020.601921 (PMC7861041; doi:10.3389/fmicb.2020.601921)
Supplement: Supplementary file 1 [file Image_1.pdf]

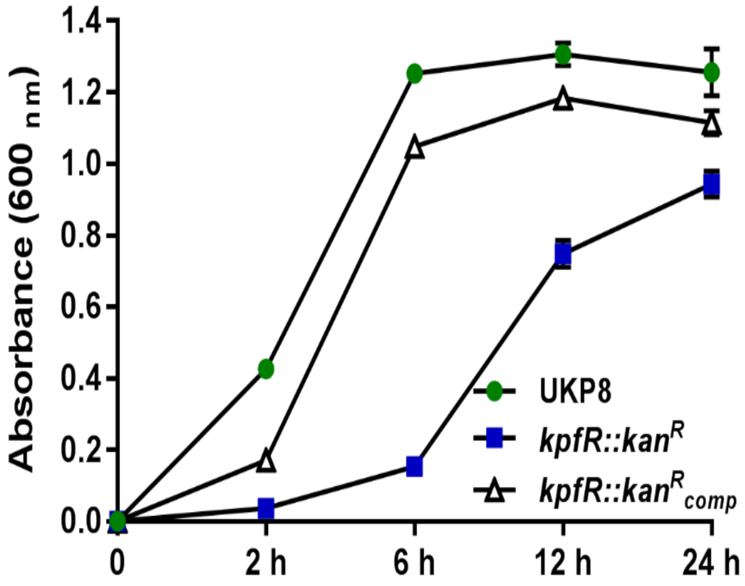

Figure S1. Growth curves of UKP8, *kpfR*::*kan*^{R} and *kpfR*::*kan*$^R_{comp}$ assessed by monitoring the optical density of the strains grown in LB medium at 37 ^oC with agitation. Despite the delayed entry into logarithmic (log) phase due to prolonged growth at lag phase, the mutant strain *kpfR*::*kan*^{R} exhibit a growth rate similar to that observed in the wild-type UKP8 on the log phase. The growth curve observed on the wild-type strain was reestablished on the complemented mutant strain *kpfR*::*kan*$^R_{comp}$.
