# Supplementary material for: Functional Insights From KpfR, a New Transcriptional Regulator of Fimbrial Expression That Is Crucial for Klebsiella pneumoniae Pathogenicity
Source: Front Microbiol. 2021 Jan 21;11:601921. doi: 10.3389/fmicb.2020.601921 (PMC7861041; doi:10.3389/fmicb.2020.601921)
Supplement: Supplementary file 2 [file Table_1.pdf]

Supplementary Table S1. Primer pairs used in PCR reactions to confirm the polycistronic transcription of *kpfR* and *kpf* gene cluster. Primer pairs *kpfR*-F and *kpfA*-R were also used to confirm the insertion of the intron in the coding region of the *kpfR* gene.

**Distance**	***Primers*[1]**	**Sequence (5' > 3')**	**Amplicon** (base pairs)
kpfR to *kpfA*	*kpfR*-F	TTCAACAATTAGCCGCACTG	845
	kpfA-R	TTAGTGATAGGCGCCTCGTT	
kpfA to kpfD	*kpfA*-F	GCCAGAAGTTGGGGTCAAT	3773
	kpfD-R	TGCTGGAGACTTTAATGATGCTC	

1. F, forward primer; R, reverse primer.
