# Supplementary material for: Functional Insights From KpfR, a New Transcriptional Regulator of Fimbrial Expression That Is Crucial for Klebsiella pneumoniae Pathogenicity
Source: Front Microbiol. 2021 Jan 21;11:601921. doi: 10.3389/fmicb.2020.601921 (PMC7861041; doi:10.3389/fmicb.2020.601921)
Supplement: Supplementary file 3 [file Table_2.pdf]

Supplementary Table S2. The most efficient insertion site on *kpfR* coding region predicted by TargeTron Design algorithm.

**Position[1]**	**Target sequence**	***Score*[3]**	***E-value*[3]**
495-496	CATCATATTGCTAAGGTCGGTGGCCTGAGC-***intron***-AATTTTCTTATTATT	9.52	0,034

1. Nucleotide position of the RNA intron insertion at the antisense strand of *kpfR* gene.

2. Nucleotide sequence of the insertion site within *kpfR* gene.

3. *Score* and *E-value* as calculated by TargeTron Design algorithm.
