# Supplementary material for: Functional Insights From KpfR, a New Transcriptional Regulator of Fimbrial Expression That Is Crucial for Klebsiella pneumoniae Pathogenicity
Source: Front Microbiol. 2021 Jan 21;11:601921. doi: 10.3389/fmicb.2020.601921 (PMC7861041; doi:10.3389/fmicb.2020.601921)
Supplement: Supplementary file 4 [file Table_3.pdf]

Supplementary Table S3. Nucleotide sequence of the primers IBS-*kpfR*, EBS1d-*kpfR* and EBS2-*kpfR* primers generated by TargeTron design algorithm. These primers were used in PCR reactions to retarget the RNA segment of the intron.

Primers	**Nucleotide sequences (5’>3’)**
IBS-*kpfR*	AAAAAAGCTTATAATTATCCTTAGGTGGCCTGAGCGTGCGCCCAGATAGGGTG
EBS1d-*kpfR*	CAGATTGTACAAATGTGGTGATAACAGATAAGTCCTGAGCAATAACTTACCTTTCTTTGT
EBS2-*kpfR*	TGAACGCAAGTTTCTAATTTCGATTCCACCTCGATAGAGGAAAGTGTCT
