# Supplementary material for: Functional Insights From KpfR, a New Transcriptional Regulator of Fimbrial Expression That Is Crucial for Klebsiella pneumoniae Pathogenicity
Source: Front Microbiol. 2021 Jan 21;11:601921. doi: 10.3389/fmicb.2020.601921 (PMC7861041; doi:10.3389/fmicb.2020.601921)
Supplement: Supplementary file 5 [file Table_4.pdf]

Supplementary Table S4. Primer pairs used to amplify the entire *kpfR* coding region plus 3' and 5' flanking regions to generate the complemented strain.

Primers	**Sequence (5'>3')**	**Tm**	**Amplicon** (base pairs)
kpfR-F_{com}	AGCTAAGCTTCAGCCCGAG	60º C	2426
kpfB-R_{com}	GCTGCCACTGCAGATTTTC		

The forward primer (*kpfR*-F_{com}) contains a restriction site for *Hind*III (underlined nucleotides). The PCR product was digested with *Hind*III and *Kpn*I restriction enzymes, rendering a digested fragment of 1825 nucleotides comprising the entire coding region of *kpfR* gene plus 568 nucleotides upstream the ATG start codon of *kpfR* and 555 nucleotides downstream the stop codon TAA of *kpfR*. The digested fragment was cloned on pCR2.1-TOPO vector, and the recombinant vector was used to transform the *kpfR*::*kan*^{R} mutant strain to generate the complemented strain.
