# Supplementary material for: Functional Insights From KpfR, a New Transcriptional Regulator of Fimbrial Expression That Is Crucial for Klebsiella pneumoniae Pathogenicity
Source: Front Microbiol. 2021 Jan 21;11:601921. doi: 10.3389/fmicb.2020.601921 (PMC7861041; doi:10.3389/fmicb.2020.601921)
Supplement: Supplementary file 6 [file Table_5.pdf]

Supplementary Table S5. Primer pairs used on RT-qPCR reactions. Primers were designed using *Primer3 version 4.1.0* web-program, in order to present 60 ^{o}C of annealing temperature and amplicon sizes ranging from 95 to 105 base pairs (bp).

**Genes** (cluster)	***Primers***	**Sequence (5'-3')**	**Amplicon** (bp)
kpfR [1] (*kpf* cluster)	*kpfR*-F<br>*kpfR*-R	TTCAACAATTAGCCGCACTG<br>ACTGGATCGGCAAGAATCTC	95
kpfA (*kpf* cluster)	*kpfA*-F<br>*kpfA*-R	GCCAGAAGTTGGGGTCAAT<br>TTAGTGATAGGCGCTTCGTT	99
fimA (*fim* cluster)	*fimA*-F<br>*fimA*-R	CGTCGGTTTCAACATCCAG<br>GGTGGTATTGCTGCTGTCG	100
ecpA (*ecp* cluster)	*ecpA*-F<br>*ecpA*-R	AATATTATGGGCGGCAACCT<br>CCGCTGATGATGGAGAAAGT	98
mrkA (*mrk* cluster)	*mrkA*-F<br>*mrkA*-R	GGCTGCTGGTGGCACTAAA<br>GCCAGGTAGCCCTGTTGTT	105
galF (*cps* cluster)	*galF*-F<br>*galF*-R	GCGAATTTGAAAGCGGTTATT<br>TGAATCATTGGCTTATCAACGA	110
rho (endogenous control)	*rho*-F<br>*rho*-R	AACTACGACAAGCCGGAAAA<br>ACCGTTACCACGCTCCATAC	99

1. This primer pair anneals in the *kpfR* gene at a position upstream to the RNA intron insertion site on *kpfR*.
